# The Interplay Between Oxidant/Antioxidant System, Transcription Factors, and Non-Coding RNA in Lung Cancer

**DOI:** 10.3390/ijms26167679

**Published:** 2025-08-08

**Authors:** Caterina Di Sano, Claudia D’Anna, Angela Marina Montalbano, Mark Gjomarkaj, Mirella Profita

**Affiliations:** Institute of Translational Pharmacology—National Research Council of Italy (IFT-CNR), 90146 Palermo, Italy; caterina.disano@cnr.it (C.D.S.); claudia.danna@cnr.it (C.D.); angelamarina.montalbano@cnr.it (A.M.M.); mark.gjomarkaj@cnr.it (M.G.)

**Keywords:** lung cancer, Nc-RNA, oxidative stress, transcription factors

## Abstract

The exposure to risk factors, such as cigarette smoke and air pollution (containing metabolic oxidants and toxic substances), leading to cellular and molecular alterations, promotes the development of lung cancer at multiple stages. The antioxidant defence system plays a critical role in counteracting the mechanisms of oxidative stress. In physiological conditions, the balance between pro-oxidant and antioxidant species is critically important for the correct performance of cellular functions. Its imbalance is accompanied by the onset and progression of various pathologic states, including lung cancer. Cell signalling pathways and non-coding RNAs play a crucial role in the mechanisms of carcinogenesis and in the development of resistance to conventional therapeutic treatments. The interplay between the oxidant/antioxidant system, transcription factors, and non-coding RNAs is involved in the development and in the pathogenesis of lung cancer. This review provides a comprehensive resource for researchers and clinicians to better understand this intricate system and its cellular interactions, with the aim of disseminating the knowledge of the mechanisms involved in both cancer development and the development of new anti-cancer therapeutic strategies. A thorough understanding of the interplay between oxidative stress mechanisms, the activity of transcription factors, and non-coding RNAs could improve the efficacy of drug treatments and open new pharmacological perspectives for the control of inflammation and disease progression in lung cancer.

## 1. Introduction

Lung cancer is one of the most prevalent and lethal cancers, with a rather low five-year survival rate that varies significantly depending on several factors, including the stage of the disease at the time of diagnosis, the specific characteristics of the tumour, and the therapies used. Indeed, despite advances in early diagnosis and targeted therapeutic strategies, the overall prognosis remains bleak for many patients with lung cancer, particularly non-small cell lung cancer (NSCLC) [1]. The pathogenesis of lung cancer is the result of a complex interaction between genetic mutations, environmental factors (primarily tobacco smoke), and alterations in signalling pathways. This highlights the need for an ever more in-depth analysis of the mechanisms that regulate the onset and evolution of the disease [2]. Elements such as oxidative stress, transcription factors (TFs), and non-coding RNAs (ncRNAs) play a key role in regulating gene expression and influencing the onset, development, metastasis, and treatment resistance of lung cancer. Their potential use as biomarkers for early diagnosis, prognostic evaluation, and monitoring of therapeutic response is increasingly recognised in lung cancer [3,4]. Current perspectives suggest that targeting oxidative stress, modulating the activity of TFs, and manipulating ncRNAs might constitute an innovative and promising approach for the development of new therapeutic strategies against lung cancer [5].

Genetic changes are responsible for alterations underlying tumorigenesis and involve ncRNAs and ROS. Oxidative stress regulates every stage of tumour development [6]. The interaction between ROS and ncRNAs regulates multiple genes and pathways involved in various forms of cancer, including lung cancer. NcRNA-ROS regulate several cancer-related cell signalling pathways, such as nuclear factor kappa-light-chain-enhancer of activated B cells (NF-κB), nuclear factor erythroid 2-related factor 2 (Nrf2), p53 phosphatase tensin homolog (PTEN), and the wingless (Wnt)/glycogen synthase kinase-3 beta (GSK3β) integration site. The mechanism of this relationship has not yet been fully elucidated. To date, most studies on the relationships between ncRNAs, oxidative stress, and carcinogenesis are based on cell lines and in some cases on animal model studies [7]. Preclinical cell models play a fundamental role in understanding the cellular and molecular mechanisms underlying humoral transformation of cells and their response to pharmacotherapy [8]. Many tumour studies involve two-dimensional (2D) monolayer cultures, despite limitations due to the inability to simulate the complexity of the tumour microenvironment and dynamic changes in cell–cell interactions [9]. However, three-dimensional (3D) culture models are able to reproduce phenotypic and molecular characteristics of the primary tumour. They represent a resource for studying tumour growth, invasion, and therapeutic response [10]. However, lung cancer research in 3D models is limited, and 2D and 3D culture systems often increase the complexity of our preclinical research tools by providing information on NSCLC cells, helping to transfer treatment strategies from the laboratory to the clinic. Accordingly, it was reported that Plasma-activated medium (PAM) has anti-tumour characteristics in two-dimensional (2D) and three-dimensional (3D) cultures of A549 cells (non-small cell lung cancer cell line). These preclinical models showed that the treatment of the cells with PAM downregulated the expression of the RAS/ERK signalling pathway, inhibiting the viability, migration, and invasion abilities and epithelial–mesenchymal transition (EMT) process in both 2D and 3D cultures, as well as the tumour spheroid formation [11].

The primary goal of this review is to broaden and improve understanding of the mechanisms involved in tumorigenesis, contributing to the identification of new strategies for the development of new anticancer treatments. We aim to provide a comprehensive resource for researchers and clinicians to understand and address the complex interactions of the oxidant/antioxidant system with the activation of transcription factors and related ncRNAs in lung cancer. A thorough analysis of the molecular mechanisms underlying these factors is essential for developing increasingly targeted and effective diagnostic and therapeutic strategies, with the goal of improving patient survival and quality of life. Therefore, it is important to explore each of these components and their impact on lung cancer. We have reported the most recent published data to broaden the understanding of the role of the oxidant/antioxidant system, transcription factors, and ncRNAs in lung cancer.

## 2. Oxidant/Antioxidant System

Oxidative stress takes part in processes ranging from tumorigenesis to tumour death via a variety of pathways and processes, including mitochondrial stress, endoplasmic reticulum stress, and ferroptosis. Oxidative stress can affect cell fate by engaging in the complex relationships between senescence, death, and cancer. The oxidant/antioxidant system plays a crucial role in the beginning of cell damage, progression and treatment of lung cancer [12]. Indeed, the establishment of an imbalance between the production of reactive oxygen species (ROS) and the ability to neutralise them through the antioxidant defence system may favour the development of genetic mutations involved in cancer transformation, tumour progression, metastasis production, and chemoresistance. ROS are highly reactive molecules that include free radicals, such as superoxide (O_2_−), hydroxyl radicals (OH), and non-radiative species such as hydrogen peroxide (H_2_O_2_) [13,14]. At the cellular level, they are produced by a variety of sources, including NADPH oxidases, phase I metabolizing enzymes of the cytochrome P450 system, and the mitochondrial electron transport chain (ETC) [15]. The P450 system employs molecular oxygen to generate superoxide anions and other ROS, which are involved in cellular signalling processes or may lead to oxidative stress and tissue injury [16]. NADPH oxidases are membrane-associated enzymes, which produce superoxide in response to signalling pathways triggered by inflammatory stimuli. These enzymes are essential for immune defense, producing ROS to eliminate pathogens; however, prolonged oxidative activity promotes chronic conditions in the disease. Nevertheless, mitochondria, through the electron transport chain (ETC), are the main source of oxidative stress. These mechanisms play a key role in controlling ROS levels, thereby preventing oxidative stress and safeguarding tissues from damage. Additionally, environmental stressors, such as infections, cigarette smoke, and aging, can induce oxidative stress through the activation of macrophages and neutrophils, or by reducing mitochondrial enzymatic activity [17].

The components of oxidative stress, especially ROS, act as cellular messengers within the mitochondria and normally take part in the control of pathways involved in the cell metabolism and growth. They are involved in the regulation of various cellular functions, such as the immune response, the cell cycle, and cell signalling pathways [18]. An overproduction of ROS can cause damage to DNA, proteins and lipids, leading to cellular dysfunctions that in turn contribute to cancer development [19]. High levels of ROS are oncogenic agents generating mutation of healthy cells in tumour cells. They can cause mutations in genes crucially related to tumour suppressors (e.g., p53) and protooncogenes (e.g., KRAS, EGFR), contributing to the initiation of lung cancer by promoting activation of signalling pathways and genetic alterations involved in cell cycle malfunction, resistance to apoptosis, and tumour progression. This was observed in various cancer cell line cultures from different tissues, such as pancreas, breast, skin, and lung [20]. Mutant p53 Isoforms damage the antioxidant system, driving a pro-tumorigenic survival in cancer cells [21].

Oxidative stress promotes tumour growth and spreads through various processes. It activates TFs such as NF-κB and affects the release of pro-inflammatory cytokines and growth factors, including vascular endothelial growth factor (VEGF). VEGF initiates the process of angiogenesis by influencing the gene expression of biomarkers involved in the regulation of adhesion, migration and invasion of lung cancer cells. All these functions promote the spread of tumour cells from the primary tumour to distant organs and the resistance to chemotherapy, thus increasing their survival during the treatment of lung cancer [22,23].

A complex three-line antioxidant system is physiologically capable of neutralising ROS and repairing oxidative damage, thus protecting cells from oxidative stress [24]. The first line of defences regulates the dismutation of superoxide radicals (O_2_^−^) and hydrogen peroxide (H_2_O_2_), removes superoxide radicals and prevents the formation of the more harmful peroxy-nitrite ONOO_2_−, maintaining the physiological level of nitric oxide (NO_2_) important for normal cellular metabolism. It includes enzymes such as (1) superoxide dismutase (SOD) involved in the conversion of superoxide radicals into hydrogen peroxide; (2) catalase (CAT) that converts hydrogen peroxide into water and oxygen, preventing cellular damage; (3) glutathione peroxidase (GPx) that reduces hydrogen peroxide and organic peroxides using glutathione (GSH) as a substrate; and (4) thioredoxin reductase that maintains the reducing environment within cells and is involved in detoxification of ROS [25]. A second line of defence involves exogenous non-enzymatic antioxidants and small molecules, such as vitamins C and E (ascorbate and alpha-tocopherol, respectively, essential for protection against ROS, as they donate electrons to neutralise free radicals and prevent lipid peroxidation), carotenoids (e.g., β-carotene, lycopene, lutein), flavonoids (e.g., quercetin, anthocyanins, epicatechin), and GSH (helps to remove ROS and to maintain redox balance), which, by the activation of TFs, such as Nrf2, promote protection against chronic diseases [26]. The oxidant/antioxidant system is often altered in cancer, with an increase in ROS production and a reduction in antioxidant capacity. In many lung cancer cells, antioxidant defences are frequently overwhelmed or dysfunctional, creating a chronic state of oxidative stress. This imbalance contributes to cancer progression by promoting inflammation, cell survival and resistance to apoptosis, thus allowing cancer cells to survive and proliferate. Accordingly, cancer cells produce high concentrations of ROS due to mitochondrial dysfunction, changes in metabolism, or exposure to environmental carcinogens such as cigarette smoke [13,27,28].

**Figure 1:**
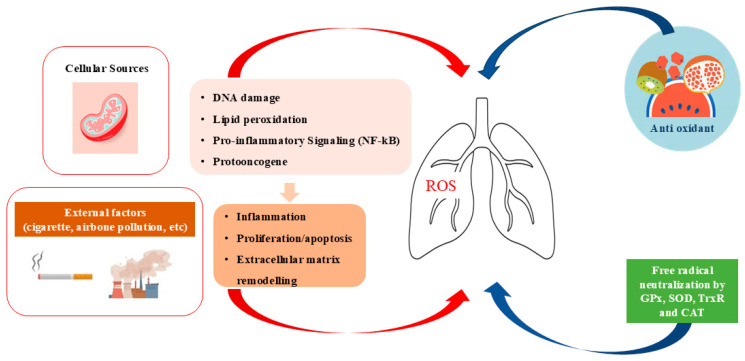
Schematic representation of the effects of oxidant/antioxidant imbalance on the lung.

ROS are particularly detrimental as they induce DNA damage, leading to genomic instability, transcriptional errors, and permanent mutations closely associated with the process of carcinogenesis, affecting DNA strand breaks, base modifications, chromosomal translocations, and covalent DNA-protein adduct formation. When DNA repair mechanisms are insufficient or overwhelmed, these lesions may become irreversible, potentially driving oncogenic transformation. Recent studies have shown that, in the A549 cell line, ROS up-regulate cadherin-3 (CDH3), a cell adhesion protein associated with cancer metastasis and poor prognosis [29]. Furthermore, ROS facilitate DNA damage in malignant cells and by the activation of apoptotic pathways, thereby contributing to the suppression of tumorigenesis disrupting tumor cell proliferation and survival. The cytotoxic effect of markers of oxidative stress was observed in cancer cells in an in vitro model of Calu-6 and A549 human lung cancer cell lines exposed to hydrogen peroxide (H_2_O_2_), showing a significant growth inhibition caused by increase in ROS levels [30,31]. Accordingly, in vivo studies demonstrated that quercetin can induce apoptosis in Lung Cancer Cells of rats and in A549 and H69 human lung cancer cell lines, reducing MDA and increasing SOD and GSH-Px levels [32] (Figure 1). All these findings might underscore the relevance of ROS modulation as a potential therapeutic strategy targeting cancer cells. These observations warrant further investigation into ROS-based interventions in oncological treatment frameworks for the lung. The limitation of these studies is that they were performed using cell line cultures (A549, Calu-6, and H1299 cell lines), not fully reproducing the microenvironment where cancer cells grow in vivo. Although ROS-modulating strategies demonstrate promising antitumor activity, their application remains confined to preclinical models, and clinical translatability has to be validated; furthermore, the absence of clinically approved ROS-based therapies and reliable predictive biomarkers poses significant challenges to their implementation in clinical settings [33]. Further clinical studies or meta-analyses regarding therapeutic targeting of oxidative stress in lung cancer might strengthen the translational relevance of these observations in 2D or 3D in vitro models.

## 3. Transcription Factors in Lung Cancer

Several cellular and molecular mechanisms are involved in the development of lung cancer, and, among them, several TFs play a key role in neoplastic progression. These factors regulate the expression of genes that influence cell growth, survival, ability to metastasise, and resistance to chemotherapy treatments. Some proteins are able to bind to specific DNA sequences, activating or inhibiting the expression of genes, thereby regulating these crucial biological processes [34]. The main TFs involved in lung cancer progression are Nuclear Factor kappa-light-chain-enhancer of activated B cells (NF-κB), p53, Hypoxia-inducible factor 1 (HIF-1), MYC, and Activator Protein-1 (AP-1), and their abnormal activation or loss of normal function can promote cancer development and spread [34]. NF-κB is a transcription factor involved in inflammatory responses, immune responses and maintenance of cell viability. It is frequently overexpressed or abnormally activated, contributing to tumour progression, metastasis formation and resistance to therapy in lung cancer cells. It can be activated by oxidative stress, promoting the production of cytokines and growth factors involved in tumour expansion and invasion of surrounding tissues. NF-κB also regulates the expression of genes that facilitate the survival of cancer cells, particularly those that might otherwise be damaged or eliminated by the immune system, helping them to escape apoptosis and allowing them to survive and proliferate out of control. Thus, in lung cancer, NF-κB activation contributes to the development of a pro-tumour microenvironment, inducing immune cell recruitment, remodelling of the extracellular matrix and resistance to apoptosis. This pro-inflammatory environment not only supports the survival of cancer cells but also contributes to escape from immune surveillance [35,36]. Finally, Nrf2 regulates the antioxidant action of thioredoxin reductase 1 (TXNRD1) and glutathione reductase (GSR) (enzymes involved in the reduction of the antioxidant protein thioredoxin (TXN)), and the antioxidant tripeptide glutathione (GSH) during lung tumour initiation and progression [37].

p53 is an onco-suppressor and is often referred to as the ‘guardian of the genome’. It is a transcription factor that regulates the cell cycle, the response to DNA damage, the induction of apoptosis and the cellular response to oxidative stress through the activation of antioxidant defence mechanisms [38,39]. When DNA damage occurs, p53 is activated to arrest the cell cycle, allowing the cell to repair the damage or, if the damage is irreparable, to trigger apoptosis, preventing damaged cells from proliferating. p53 is one of the most commonly mutated genes in lung cancer. Its mutation or inactivation leads to incorrect cell cycle control and increased susceptibility to tumour growth, as it allows tumour cells to escape cell cycle control, favouring the proliferation of damaged or abnormal cells [39]. In uncontrolled tumour growth the ability of p53 to induce apoptosis is compromised, and tumour cells’ resistance to DNA damage signals. Furthermore, mutation of p53 impairs its ability to handle oxidative stress, leading to uncontrolled ROS production and promoting tumorigenesis. Loss of p53 function is often associated with a poorer prognosis, as it also contributes to resistance to chemotherapy treatments, as its inability to activate apoptosis reduces the efficacy of many DNA damage-inducing therapies. Therefore, p53 mutation is an important event in lung carcinogenesis and is a key factor in lung cancer progression and resistance [40].

HIF-1 is a transcription factor activated in response to hypoxia conditions. Under normal conditions, oxygen levels are sufficient to maintain cellular equilibrium. Lung cancer cells grow rapidly to create a hypoxic environment and to activate the overexpression of HIF-1, which controls the expression of genes involved in the process of angiogenesis to favour the invasion, spread and metastasising of tumour cells [41,42]. HIF-1 activation generates oxygen-deprived cells less sensitive to chemotherapeutic agents, reducing the effectiveness of tumour treatments [43,44]. HIF-1 also promotes tumour cell motility and invasiveness by modulating the expression of genes involved in extracellular matrix degradation and epithelial–mesenchymal transition (EMT). Additionally, it regulates the expression of TWIST, SNAIL, and ZEB1 transcription factors involved in the EMT. These inhibit the expression of E-cadherin, a molecule crucial for cell adhesion, and induce a more migratory and invasive mesenchymal phenotype. Another target gene of HIF is CXCR4, a chemokine receptor that drives the migration of tumour cells to specific target organs, such as lung, liver or bone marrow, in response to its ligand CXCL12, making CXCR4 a key element in distant metastatic colonisation [45,46]. Furthermore, HIF induces transcription of the matrix metalloproteinases MMP2 and MMP9, enzymes capable of degrading extracellular matrix components, thus facilitating invasion of the surrounding tissue [47]. Finally, HIF promotes the activation of the c-Met receptor for HGF (hepatocyte growth factor), implicated in cell proliferation, motility and survival, further contributing to tumour aggressiveness [48].

MYC is an oncogenic transcription factor involved in cell proliferation and metabolism. It is known to play a key role in cell cycle regulation and activation of genes involved in cell proliferation. In lung cancer, its overexpression is associated with disease progression. MYC inhibits cell differentiation and promotes uncontrolled proliferation of tumour cells by preventing their specialization and limiting their ability to proliferate. Consequently, the cells remain in an immature, state showing an uncontrolled growth, contributing to their expansion and tumour growth. The MYC oncogene is frequently deregulated in both non-small-cell lung cancer (NSCLC) and small-cell lung cancer (SCLC), contributing to uncontrolled proliferation, metabolic plasticity and tumour immune evasion [49]. MYC promotes invasiveness of cancer cells in SCLCs and NSCLCs, facilitating their ability to migrate and invade surrounding tissues by enhancing VEGF and TNF-β production [50]. Accordingly, under stressful conditions, it inhibits apoptosis allowing cancer cells survival and favouring chemoresistance. Finally, MYC affects cell metabolism by promoting anaerobic conditions, low-oxygen environments involved in the carcinogenesis in the lung [51,52]. Finally, a recent study shows that MYC affects glycolysis in stimulating a rapid cell growth and metastasizing in a panel of SCLC cell lines [53].

**Figure 2:**
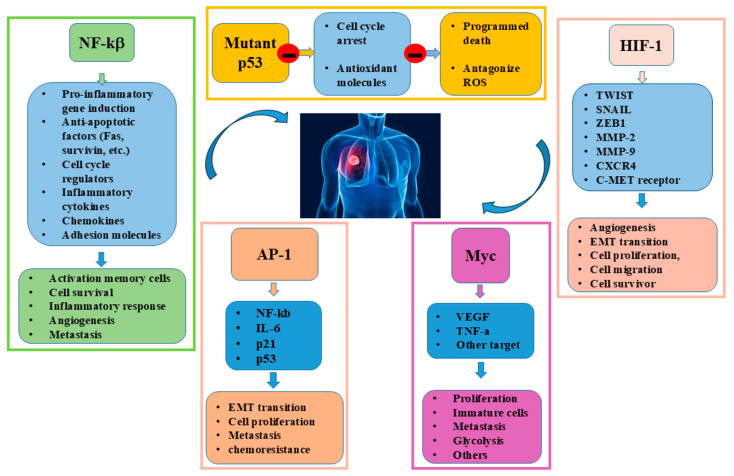
Role of Trascription Factors in lung carcinogenesis.

AP-1 is a complex of TF that regulates numerous cellular processes, including cell proliferation, differentiation, stress response, inflammation and cell death. It is composed of various members of the Jun, Fos, ATF, and MAF families of proteins, that by binding to DNA activate or suppress the expression of specific genes. NF-kB, IL-6, p21, and p53 represent some of their downstream targets. Abnormal activation of AP-1 is associated with lung cancer progression and metastasis, as it plays a crucial role in lung cancer biology, influencing proliferation, invasion, metastasis, and resistance to treatment, contributing to tumour progression and to the creation of an environment conducive to cancer cell survival [54]. For instance, c-Fos is involved in the onset of NSCL, promoting EMT progression, and it has been demonstrated that, in lung adenocarcinoma, the aberrant regulation of the Notch-1/AP-1/miR-451 axis is responsible of chemoresistance (Figure 2).

NSAIDs, such as aspirin, sodium salicylate, and sulindac, inhibit NF-κB by preventing IκB degradation, thereby blocking its nuclear translocation and enhancing TNF-mediated apoptosis. Similarly, drugs like doxorubicin, vinblastine, and vincristine promote IκB phosphorylation and degradation via protein kinase C in A549 lung cancer cells [55]. Despite these advances in the preclinical model, the broader landscape of TFs in lung cancer remains complex and only partially understood, with context-dependent functions and regulatory interactions that still limit their therapeutic targeting. A deeper understanding of the mode of action of promising inhibitors/target-specific agents needs to be further explored in future studies to gain new insights into the design or development of drugs that effectively inhibit TF activity in lung cancer.

## 4. Role of nc-RNAs in Lung Cancer

Nc-RNAs are RNA molecules that do not transcribe into proteins; however, they play a crucial and regulatory function at the transcriptional and post-transcriptional levels during gene expression. They regulate cellular processes as differentiation, proliferation, migration, apoptosis, and immune responses. NcRNAs are involved in lung cancer progression, influencing tumorigenesis (cell growth, metastasis and resistance to therapeutic treatments) [56]. There are different classes of ncRNAs: miRNAs, lncRNAs as well as piwi-interacting RNAs (piRNAs) and small ncRNA, both involved in carcinogenesis [57].

### 4.1. MiRNA

MiRNAx are small nc-RNA molecules (approximately 22 nucleotides) that regulate post-transcriptional gene expression by binding to the 3′ untranslated region (UTR) of target messenger RNAs (mRNAs), leading to their degradation or inhibition of translation. In lung cancer, miRNAs can act either as tumour suppressors or as oncogenes (oncomiR) depending on their target genes. The miRNAs that act as onco-suppressors, such as miR-34a, miR-143, and miR-145, are frequently downregulated in lung cancer [58]. In contrast, miRNAs acting as oncogenes, such as miR-21, also regulate the tumour microenvironment, influencing the production of cytokines, growth factors and extracellular matrix proteins affecting cell–cell communication [59]. In lung cancer, miR-21 and miR-210, can be secreted into the extracellular environment, favouring the escape from the immune system’s surveillance trough the modulation of the expression checkpoint molecules [60].

### 4.2. LncRNAs

LncRNAs are longer ncRNAs (up to 200 nucleotides) involved in chromatin remodelling, gene silencing, and transcriptional regulation. Their ability to influence various cellular mechanisms is involved in numerous aspects of the tumour biology of lung cancer [61]. LncRNAs alter the physical and biochemical properties of the tumour microenvironment, influencing the mechanisms of cell–cell interactions between tumour cells and immune cells. [62]. The activity of lncRNAs is associated with the development and progression of NSCLC. It was observed that the upregulation of H19 enhances STAT3 expression and modulates miR-17, promoting NSCLC cell growth, invasion, and migration. Similarly, the overexpression of ANRIL silences KLF2 and P21 to promote proliferation and to inhibit apoptosis by recruiting EZH2, ultimately contributing to poor prognosis in NSCLC [63].

Metastasis-Associated Lung Adenocarcinoma Transcript 1 (MALAT1) is another lncRNA overexpressed in lung cancer. It is involved in the regulation of the tumour microenvironment by modulating the remodelling of the extracellular matrix and influencing the migration and invasion of tumour cells. It can also regulate the secretion of growth factors and cytokines that affect communication between tumour cells and surrounding stromal cells. Another lncRNA overexpressed in lung cancer and involved in tumour progression is HOTAIR (HOX Transcript Antisense Intergenic RNA), which modulates the immune response to the tumour and influences the polarisation of macrophages towards the pro-tumour M2 phenotype; this results in an immunosuppressive microenvironment that promotes tumour growth [62,64].

MiRNAs and lncRNAs can also be found sequestered within vesicles called exosomes. Exosomes are small lipid vesicles, between 30 and 150 nm in size, released by cells into their extracellular environment. These vesicles contain a variety of molecules, including proteins, lipids, and mRNAs, which can be transported from one cell to another, facilitating cell communication [65]. Exosomes facilitate communication between lung cancer cells and their surrounding microenvironment by transporting biologically active cargo to transfer genetic information to neighbouring cells. In this manner, cancer cells change their behaviour to play a key role in intercellular communication that influences tumour progression, metastasis and drug resistance by promoting processes such as angiogenesis, epithelial–mesenchymal transition (EMT)**,** and immune evasion [66,67,68].

### 4.3. CircRNAs

Circular RNAs (circRNAs) represent a class of ncRNAs characterised by a covalently closed loop structure, giving them greater stability than linear RNAs. Some circRNAs contain open reading frames (ORFs) and can be translated into functional peptides implicated in the development and progression of different tumour types. In lung tumorigenesis, circRNAs represent a new class of molecular regulators involved in the regulation of several mechanisms that modulate growth, invasion and resistance to drug treatment expression [69]. Homeodomain-interacting protein kinase (ircHIPK3) is one of the circRNAs most commonly studied in the oncological context. It is frequently overexpressed in NSCLC tissue, playing an oncogenic role by sequestrating the onco-suppressor miRNAs (miR-124, miR-149, miR-379), resulting in deregulation of key genes for tumour growth, such as CDK6 and STAT3 [70]. In vitro studies on human lung tumour cell lines A549 and H1299 have shown that IrcHIPK3 acts as a molecular sponge for miR-107, promoting tumorigenesis [71]. Furthermore, circ-IAR, secreted by exosomes, is a novel oncogene that affects the malignant development in NSCLC via the endogenous RNA regulatory axis, competing with circ-IARS/miR-1252-5p/HDGF [72].

### 4.4. PiRNA and Small nc-RNA

**Figure 3:**
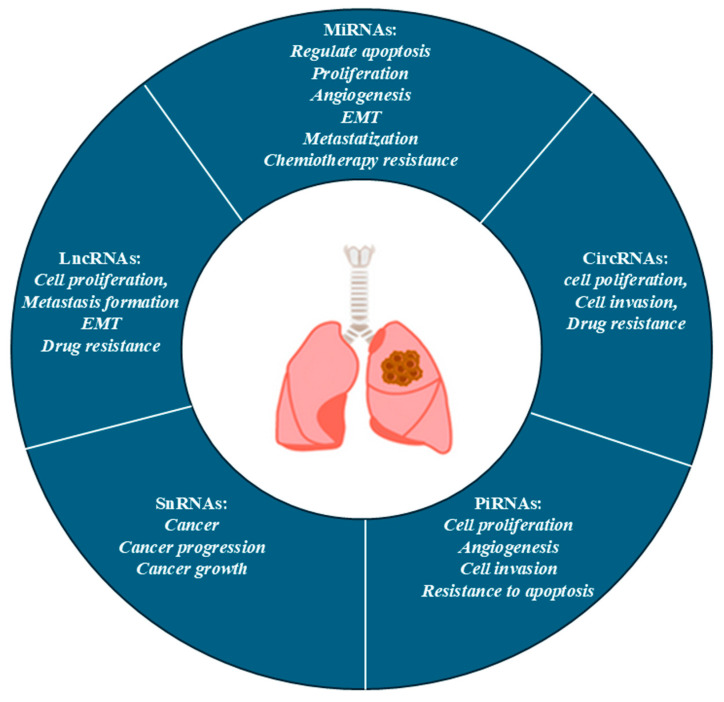
Impact of nc-RNAon lung cancer.

PIWI-interacting RNAs (piRNAs) are a class of small non-coding RNAs, typically 24–31 nucleotides long, that interact with PIWI proteins, and can be altered in lung cancer. PiRNAs can act as oncogenes or tumor suppressors depending on their expression levels. In lung cancer, specific piRNAs (e.g., piR-651, piR-55490) are linked to tumor growth proliferation, invasiveness, and poor prognosis. In particular, PiR-55490 directly regulates oncogenic pathways such as mTOR/AKT and is associated with poor prognosis, and PiR-651 has been identified as a promoter of cell proliferation and invasiveness in NSCLC, acting through the cyclin D1/CDK4 pathways. The differential expression of PiRNAs in smokers vs. non-smokers supports their potential role as a biomarker of lung cancer and suggests their relevance as diagnostic and therapeutic targets [73]. Finally, SnRNAs, such as U1, U2, U4, U5, and U6 are known to be components of the spliceosome, able to remove introns from pre-mature RNA. Mutations in genes coding for snRNAs have been observed in lung cancer, contributing to tumour progression by aberrant splicing of oncogenes or onco-suppressor genes [74,75] (Table 1, Figure 3).

Although nc-RNAs show exceptional promise as therapeutic targets and non-invasive biomarkers in lung cancer, substantial challenges remain to be achieved. Still present today are inconsistent validations across cohorts [83,84], a limited specificity, mechanistic heterogeneity, delivery limitations, and poor integration with known therapeutic resistance pathways [83,85]. In this scenario, identifying the importance of nc-RNAs in multiple cellular and molecular activities associated with the origin or progression of lung cancer through in vitro/in vivo experimental models could support new ideas for clinical trials or meta-analyses. This scientific approach could ensure the increased production of translational data that define the relevance of therapeutic targeting of ncRNAs in lung cancer.

## 5. Interplay Between Oxidative Stress, nc-RNA, and TFs in Lung Cancer

As extensively discussed, lung cancer remains a leading cause of cancer-related deaths worldwide and ROS are positively correlated with its genesis and malignant progression. As mentioned above, oxidative stress is involved in the regulation of several biological processes, activating transcription factors including NF-κB, AP1, p53, and HIF-1, thus significantly shaping tumor development [6]. At the same time nc-RNAs such HOTAIR and LncRNA-p21 respond to oxidative stress by altering their cellular abundance, thereby regulating gene expression networks involved in ROS balance, or suppressed by these same transcription factors, playing a key role in activating signalling pathways that lead to a tumor-friendly environment for metastasis and uncontrolled growth [86,87]. Nc-RNA synthesis is finely regulated by a wide variety of TFs depending on cellular context. TFs bind to specific DNA sequences and contribute to the regulation of nc-RNA transcription and synthesis, increasingly recognised as key mediators in cancer development, and their action is associated with the mechanisms of interference of cell cell communication, gene expression and modulation of the cellular microenvironment [88]. Prolonged oxidative stress causes inflammation by activating transcription factors altering gene and protein expression involved in growth factors, tumor-suppressor genes, oncogenes, and pro-inflammatory cytokines, resulting in cancer cell survival. In this manner, oxidative stress and inflammation affect the breakdown of cascade of biological signalling closely linked to cancer origin and progression [89,90]. However, despite a strong relationship between nc-RNAs t ROS creation and signalling pathway, there are few scientific studies of a clinical translational nature that simultaneously investigate these aspects in lung cancer.

### 5.1. Interplay and MYC

Lung cancer cells exhibit elevated ROS production in comparison to normal cells and, together with other regulatory mechanisms, the interaction between ROS and ncRNAs modulates the expression of various genes and pathways involved in cancer. In agreement with this statement, it was found that Myc engages a positive feedback loop with lncRNA HIF1A-As2, leading to cell proliferation and tumor metastasis in NSCLC. Moreover, c-myc/miR-150/EPG5 axis mediated dysfunction of autophagy contributes to NSCLC development, providing a potential new diagnostic and therapeutic target in NSCLC [91,92].

### 5.2. Interplay and p53

P53 regulates the synthesis of specific ncRNAs, activating or repressing their transcription in response to DNA damage, oxidative stress and other cellular stresses. Under physiological conditions, p53 promotes the expression of tumour suppressor ncRNAs, critical in the control of the cell cycle, apoptosis and DNA repair, helping to maintain cellular homeostasis by inhibiting cell cycle progression and promoting apoptosis. In lung cancer, p53 is often mutated or inactivated, thus impairing its ability to regulate ncRNAs involved in tumour suppression [93]. Furthermore, p53, following DNA damage, activates miR-34 generating cell cycle arrest and senescence, and during this process p53 also downregulates the expression of various miRNAs, including miR-17-5p, miR-106b, and miR-155 [94]. The p53 family reaches the final frontier: the variegated regulation of the dark ncRNAs that act on inflammation and angiogenesis, promoting the formation of a pro-tumour microenvironment that promotes migration, invasion and chemoresistance [87].

### 5.3. Interplay and NF-κB

ncRNAs have recently emerged as significant regulatory molecules, particularly in relation to their interaction with the NF-κB pathway: they can be transcriptional targets, such as miR-9, miR-21, miR-143, miR-146, and miR-224, and NF-κB can also drive the expression of proteins that influence miRNA transcriptional regulation. On the other hand, there are lncRNAs able to affect NF-kb activity: among these is lncRNA *NKILA*, which inhibits the activation of NF-kB, affecting IkB phosphorylation [95]. MiR-196b-5p activates the NF-κB signalling pathway in NSCLC, and generally, in lung cancer, miR-506 has been shown to directly target and suppress the NF-κB p65 subunit, leading to the production of ROS and triggering p53-mediated apoptosis [96,97].

### 5.4. Interplay and HIF-1α

HIF-1α regulates the transcription of various nc-RNAs that promote angiogenesis, adaptation to hypoxia and cell survival under low oxygen conditions. In lung cancer, HIF-1α is upregulated in response to hypoxia and regulates or is regulated by ncRNA transcription, promoting angiogenesis and the related growing tumour, and facilitating the metastatic process. In agreement, it was observed that miR-214 enhances HIF-1α expression, supporting invasion in NSCL [98], and miR-18, miR-200c, and miR-549 significantly downregulate both transcript and protein levels of HIF-1α in lung carcinoma cells. Finally, Byun et al. demonstrated that miR-200c is inversely related to hypoxia-induced responses by inhibiting HIF-1α expression in A549 and NCI-H460 cells [99].

### 5.5. Interplay of AP-1

AP-1 regulates the transcription of genes, including several ncRNAs, creating a feedback loop involved in biological processes, such as cell proliferation, differentiation, inflammatory response and apoptosis. Abnormal AP-1 expression is frequently associated with various tumours, including lung carcinoma [54]. AP-1 exerts its oncogenic effects by regulating the expression of genes affecting remodulation of the tumour microenvironment, inhibition of apoptosis, promotion of angiogenesis and evasion of the immune response. Once bound, AP-1 promotes transcription of these genes, thereby contributing to the activation of molecular circuits that promote cancer progression [100].

## 6. Conclusions

Lung cancer is one of the leading causes of cancer mortality worldwide and is a highly heterogeneous and complex disease. This complexity reflects the dynamic interplay between multiple cellular regulatory systems, that act through an intricate network of molecular signals, active at different levels, both biochemical and transcriptional, and significantly contribute to the initiation, progression and therapeutic resistance of the disease. Among these, the oxidant/antioxidant system, TF and ncRNAs stand out as critical hubs of a synergistic molecular network, crucial in the processes of cell transformation, immune evasion and tumour adaptation in lung cancer. Deepening our understanding of the interplay between redox environment, transcriptional regulation and ncRNAs represents a key strategy in deciphering the molecular mechanisms that regulate lung cancer development and evolution, and it is essential to identify new, more effective, less invasive, and personalised therapeutic strategies with the potential to improve survival rates and quality of life of patients. Indeed, targeting oxidative stress, modulating the activity of TFs, and manipulating ncRNAs might offer an integrated approach to facilitate early diagnosis, and to provide new therapeutic possibilities useful in counteracting drug resistance and tumour recurrence, representing the main challenges in the treatment of lung cancer. To achieve these goals, further research is still needed to identify new molecular biomarkers for early diagnosis and prognosis, to develop targeted therapeutic strategies, such as the use of selective antioxidants, inhibitors of transcription factors or RNA therapies (miRNA mimetics, antagomiR, siRNA), and to investigate the mechanisms that confer drug resistance and tumour recurrence. The future application of this knowledge will outline a systemic view of tumour pathogenesis and will undoubtedly improve clinical outcomes for lung cancer patients. 

## Figures and Tables

**Table 1:** ncRNAs names and phenotypes of lung cancer.

Class	ncRNA	Phenotype in Lung Cancer	Reference
**miRNA**	miR-21	Oncogenic: ↑ proliferation, migration, invasion; ↓ apoptosis; promotes EMT via PTEN/Akt/GSK3β; confers chemoresistance	[76]
**miRNA**	miR-143	Tumor suppressor: ↓ proliferation, migration, invasion; inhibits angiogenesis (in non-lung models); ↓ tumor growth in NSCLC models	[77]
**lncRNA**	MALAT1	Oncogenic: promotes metastasis via ECM remodeling, motility gene regulation, and immunomodulation (e.g., via CCL2)	[78]
**lncRNA**	HOTAIR	Oncogenic: induces M2 macrophage polarization, creating immunosuppressive TME	[79]
**circRNA**	circHIPK3	Oncogenic: sponges miR-124 → ↑ STAT3/CDK4 & CDK6 → ↑ proliferation in NSCLC	[80]
**circRNA**	circ-IARS	Oncogenic (exosomal): promotes malignancy via circ-IARS/miR-1252-5p/HDGF axis	[72]
**piRNA**	piR-651	Oncogenic: overexpressed in NSCLC; ↑ proliferation and invasion via Cyclin D1/CDK4	[81]
**snRNA**	U1, U2, U4, U5, U6	Spliceosome components: mutations/alterations → aberrant splicing of oncogenes/tumor suppressors	[82]
Legend: ↑ = increase | ↓ = decrease | EMT = epithelial–mesenchymal transition | TME = tumor microenvironment			

## Data Availability

Data for this review were identified using PubMed searches (https://pubmed.ncbi.nlm.nih.gov/) and other relevant reference articles from the search and review articles. PubMed searches included the following keywords: lung cancer, oxidative stress, transcription factors, noncoding RNA. This review focused on articles published within the last 5 years.
